# Dampness and Moisture Problems in Norwegian Homes

**DOI:** 10.3390/ijerph14101241

**Published:** 2017-10-17

**Authors:** Rune Becher, Anja Hortemo Høie, Jan Vilhelm Bakke, Sverre Bjørn Holøs, Johan Øvrevik

**Affiliations:** 1 Domain of Infection Control and Environmental Health, Norwegian Institute of Public Health, P.O. Box 4404 Nydalen, N-0403 Oslo, Norway; anja129hs@hotmail.com (A.H.H.); Johan.Ovrevik@fhi.no (J.Ø.); 2 The Norwegian Labour Inspection Authority, P.O. Box 4720 Sluppen, 7468 Trondheim, Norway; Jan.Bakke@arbeidstilsynet.no; 3 SINTEF Building and Infrastructure, P.O. Box 124 Blindern, 0314 Oslo, Norway; SverreB.Holos@sintef.no

**Keywords:** dampness, indoor air, Norwegian homes

## Abstract

The occurrence of dampness and mold in the indoor environment is associated with respiratory-related disease outcomes. Thus, it is pertinent to know the magnitude of such indoor environment problems to be able to estimate the potential health impact in the population. In the present study, the moisture damage in 10,112 Norwegian dwellings was recorded based on building inspection reports. The levels of moisture damage were graded based on a condition class (CC), where CC0 is immaculate and CC1 acceptable (actions not required), while CC2 and CC3 indicate increased levels of damage that requires action. Of the 10,112 dwellings investigated, 3125 had verified moisture or mold damage. This amounts to 31% of the surveyed dwellings. Of these, 27% had CC2 as the worst grade, whereas 4% had CC3 as the worst grade level. The room types and building structures most prone to moisture damage were (in rank order) crawl spaces, basements, un-insulated attics, cooling rooms, and bathrooms. The high proportion of homes with moisture damage indicate a possible risk for respiratory diseases in a relatively large number of individuals, even if only the more extensive moisture damages and those located in rooms where occupants spend the majority of their time would have a significant influence on adverse health effects.

## 1. Introduction

Several large population studies have shown an association between respiratory-related disease outcomes and the occurrence of moisture damage or mold inside. The presence of an actual causative link behind this association is supported by results from experimental studies that show a variety of toxic and inflammatory-related responses after exposure to microorganisms (including spores, hyphae, bacterial components, and mycotoxins) thriving in humid indoor environments. Recent studies indicate that moisture damage and mold in the main living areas of the house (living room, bedroom, etc.) is most important for the development of adverse respiratory health effects [1,2]. The extent of moisture damage also appears to be of importance for the extent of harmful outcomes in the exposed [3,4,5].

Detailed/extensive surveys of building conditions (exposure situations) that may affect the indoor environment have not been performed in Norway. Thus, we do not know the exact prevalence of moisture problems or whether their occurrence has increased in recent years. If we compare with neighboring countries with similar climate conditions and probably similar practices in house construction methods, a survey of 450 randomly selected houses in Finland, estimated that approximately 55% of all Finnish house needed repairs or closer inspection [6]. In the same range where the results from a Norwegian study from 2008 where 205 homes in mid-Norway were examined [7]. This study included self-reported observations, inspections, and measurements. There were one or more visible signs of moisture problems in 50% of the houses. Even in houses where problems with dampness were not reported, the inspectors found that 42% of the houses had indicators of moisture problems. Common indicators were damp spots, swelling or capillary uptake of water in wooden materials. These indicators were detected in 18% of the houses.

Lower estimates were reported in a Nordic study, where self-reporting from about 2900 individuals indicated a total of 27% having problems with moisture during an 8-year period. When this study looked at how many that reported water damage, visible mold, and moisture problems during the past year, the average figures were 13.4%, 6.7%, and 18%, respectively. The corresponding figures for Norway were 13.4%, 4.5%, and 16.4% [8]. In data based on the Survey of Living Conditions by Statistics Norway, even lower numbers have been reported. Here, 3–4% report damp houses (self-report), that are defined as housing where there is rot or mold in all or some of the living quarters. When comparing with other countries, previous data has indicated at that least 20% of the buildings in several European countries, Canada and the United States had one or more signs of dampness [9].

Overall, the reported estimates of moisture problems in Norwegian dwellings vary considerably. Much of this variation is most likely due to the criteria used to define dampness/moisture problems/damage. In addition, building inspections by trained professionals appear to reveal more moisture damage than reported by house owners. This then makes it difficult to compare across studies unless the classification is similar, and even then, when subjective assumptions are involved, the assumptions will involve variations. However, there is reason to believe that there are relatively many homes that may have varying degrees of moisture- or dampness problems. It is therefore important to be aware of this type of indoor environment problems, and that these problems are followed up with inspections and repairs or changes in dampness or moisture-generating activities.

The estimates up to date represent a relatively large proportion of the dwellings and it is possible that this may cause an increased risk for respiratory diseases in a relatively large number of individuals. This is relevant even if only the more extensive moisture damages and those located in rooms where occupants spend the majority of their time would have significant adverse health effects. To more accurately estimate the extent of moisture problems in Norwegian dwellings, the Norwegian Institute of Public Health (NIPH) were asked in 2015 by the Norwegian Directorate of Health to collect and process existing data based on inspections registered by the pest control company Anticimex.

## 2. Materials and Methods

The Norwegian insurance company If offers quality surveys of private dwellings for customers living in detached, duplex, semi-detached and terraced houses. The survey is offered regardless of the customers’ prior knowledge of moisture or other building related problems. The building inspection is performed by qualified building inspectors from the pest control company Anticimex, and results in an extensive report that the house owner can use for eventual measures. The report reveals signs of poor indoor air quality, moisture problems, and challenges that are associated with fire and crime safety.

Approximately 15,000 homes are checked annually. The inspection covers 120 checkpoints and includes surveys of crawl space, basement, attic (un-insulated and insulated), bathrooms, toilets, washrooms, cold rooms, kitchens, bedrooms, living room, other rooms unspecified, drainage, and foundations as well as water and sewage.

For each checkpoint, a condition class (CC) is given with a four-point scale (Table 1), where CC0 is immaculate (there are no comments) and CC1 acceptable (actions not required, building parts are worn but without measures deemed necessary). In CC2, structural elements have clear signs of damage, excessive wear, or have poor functionality with a need for remediation or the building component is estimated to have a short remaining life expectancy. CC2 can also be scored to indicate the possibility for further damage of greater consequence. CC3 indicate increased wear and damage, total malfunction requiring urgent action. To further describe moisture damage, Anticimex uses an extensive phrase library as well as free text describing present damage, suspected hidden damage or risk for future damage.

Although inspectors also note the *risk* for moisture damage, we chose to only include records relating to visible/obvious moisture damage. In the review of the material, there were also large groups of risk homes, where many may have moisture damage. If these had been included in our calculations, the percentage of moisture-damaged houses would have been higher.

The results (building data, CCs, phrases and free text) of all surveys are recorded in a database, and the analyses in this paper are built on an anonymized extract form this database. The report form has been updated and changed on several occasions. For this study, only results from the last revision of the form are included in the analysis, representing data from 10,112 homes collected in 2015, containing 160,000 phrases/descriptions.

A selection of typical signs of moisture damage that was registered:

- Recent water damage
- Leakage from pipes, porcelain etc.
- Proven/measurable moisture in building structures
- High humidity (over 60%)
- Moisture spots
- Condensation
- Mold growth, decay and/or discoloring fungi
- Musty smell
- Moisture damage from an anticipated old source for moisture
- Uneven floor after previous water intrusion
- Age in combination with physical damage/deficient construction (primarily applies to damp/moisture) if simultaneous description of moisture damage
- Damage to tiles, plates, coatings, joints, welded joints etc. in damp/wet zone
- Loose tiles in bathroom in combination with moisture indications.
- Damage to tiles, plates, coatings, joints, welded joints etc. in damp/wet zone in combination with moisture indications.
- Penetrations of water/moisture from bathroom into adjacent structures/rooms!?
- Detection of inadequate drainage outside in combination with moisture on the inside.

## 3. Results

### 3.1. Prevalence of Moisture Problems

**Figure 1:**
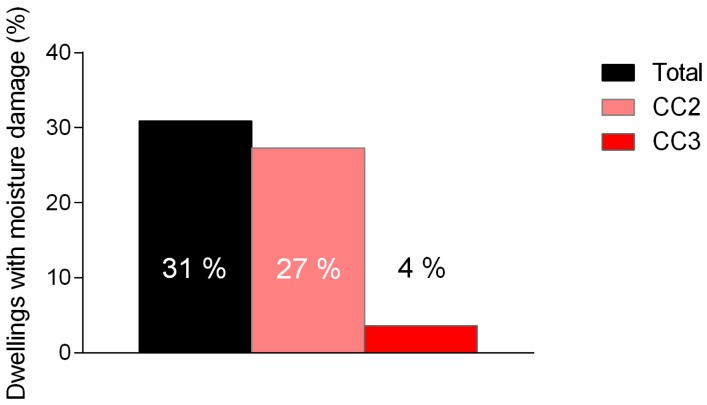
Percentage of Norwegian dwellings with different levels of moisture damage (CC2 or CC3). CC2: structural elements have clearly signs of damage, excessive wear or have poor functionality; there is a need for remediation/the building component is estimated to have a short remaining life. CC3: substantial wear and damage/total malfunction, requiring urgent action.

A total of 10,112 housing reports and an extensive list of 160,000 descriptive comments were reviewed in the present study. Of the available housing reports, 3125 homes (31%) had moisture or mold damage categorized at CC2 or CC3 levels. This amounts to 31% of the surveyed dwellings. Of these, the majority (27%) had CC2 as the worst grade in the residence, while 4% had CC 3 as the worst grade level in the residence (Figure 1).

### 3.2. Age of Houses with Moisture Problems

**Figure 2:**
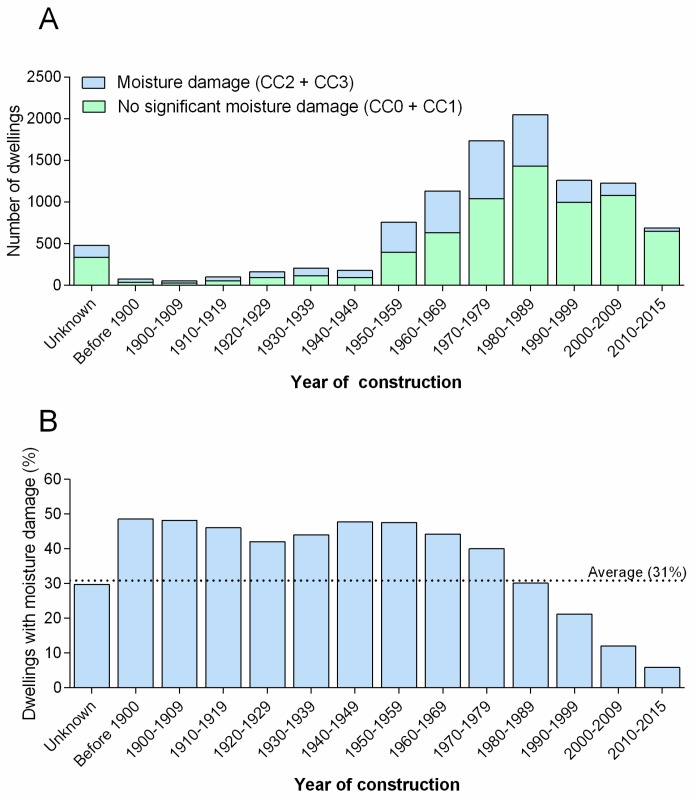
Dwellings with moisture damage distributed by year of construction. The figure shows total number of Norwegian dwellings in this study with and without moisture damage (**A**) and share of dwellings with moisture damage (**B**) grouped after year of construction.

The majority of the dwellings surveyed were built between 1950 and 2015 with the highest number from 1970 to 1989. Dwellings built before 1980 had the highest proportion of moisture damage. The share of homes with moisture damages then declined gradually the newer the dwellings were (Figure 2).

### 3.3. Geographical Area of Houses with Moisture Problems

**Figure 3:**
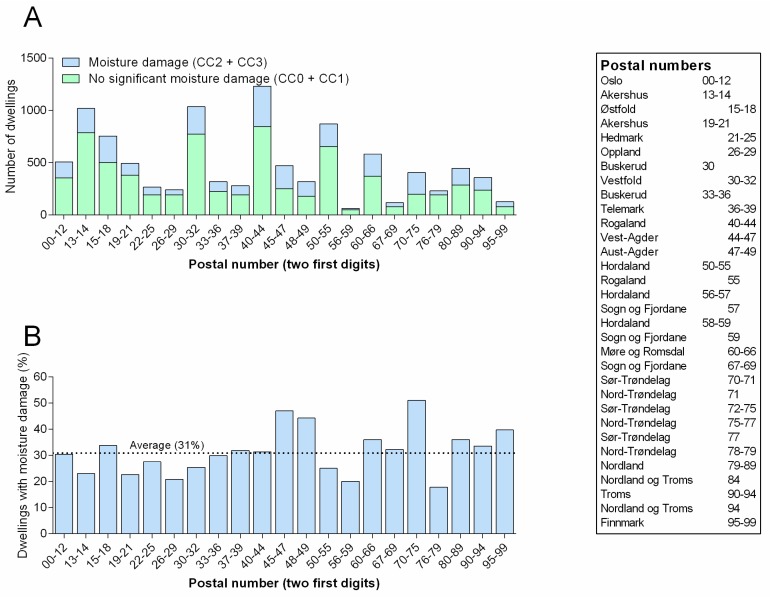
Dwellings with moisture damage distributed by geographical location. The figure shows total number of Norwegian dwellings in this study with and without moisture damage (**A**) and share of dwellings with moisture damage grouped after location/postal code (**B**).

Some geographical variations in moisture problems, ranging from 18 to 51% were reported in different regions of Norway. The southern counties West- and East Agder (Vest- og Aust Agder) and the mid Norway county of South Trøndelag (Sør Trøndelag) stand out with the highest proportion of homes with moisture damage (44%, 47%, and 51%, respectively), while North Trøndelag has the lowest (Figure 3). However, no obvious geographical pattern was observed.

### 3.4. Location of Moisture Problems in Houses

**Figure 4:**
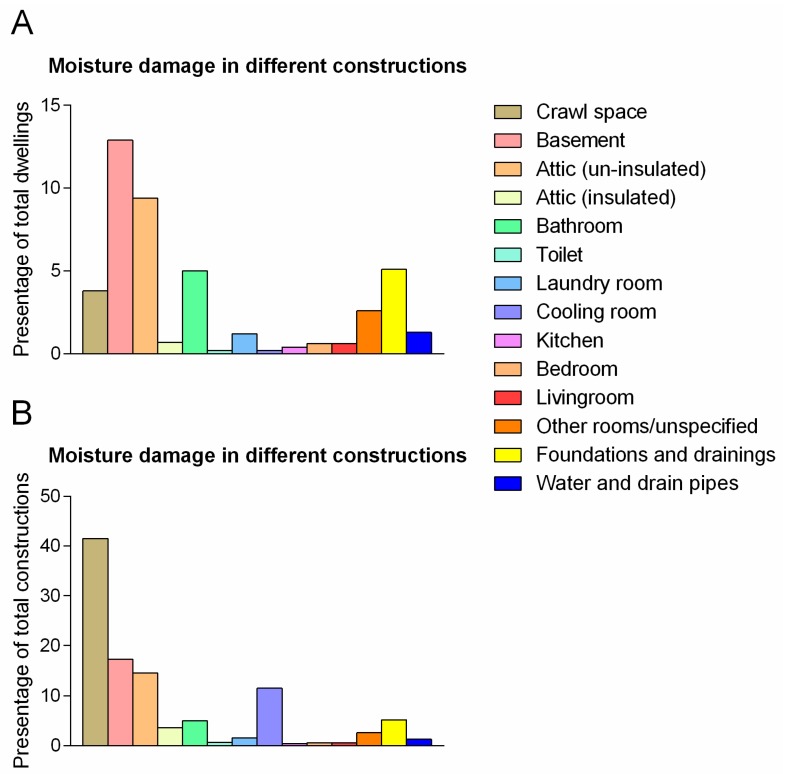
Moisture damage distributed by construction/room type. The figure display percentage of Norwegian dwellings with moisture damage in the different rooms (**A**) and the percent of the respective rooms with moisture damage (**B**). For instance, only a few inspected dwellings had crawl spaces or cooling rooms. Thus, while moisture damaged crawl spaces and cooling rooms were reported in less than 5% and 1% of all dwellings respectively, more than 40% of all crawl spaces and more than 10% of all cooling rooms were moisture damaged.

The building parts having the largest total numbers of moisture damages were in rank order: basements, unfurnished attics, foundations and drainage, bathrooms, and crawl spaces (Figure 4A). Notably, 41.5% of all crawl spaces had moisture problems, but these constituted only 4% of all the inspected homes, as only some houses had crawl spaces. Thus, adjusting for the relative occurrence of different rooms and constructions, the building parts with highest proportion of moisture problems (CC2 and CC3) are crawl spaces, basements, attics (unfurnished), cooling/refrigerated rooms (cold rooms for storing food), and areas in association with foundations and drainage (Figure 4B).

Moisture problems in living room made up less than 1%, the same as found in bedrooms and kitchens (Figure 4A).

### 3.5. Distribution of Moisture Problems Related to CC

**Figure 5:**
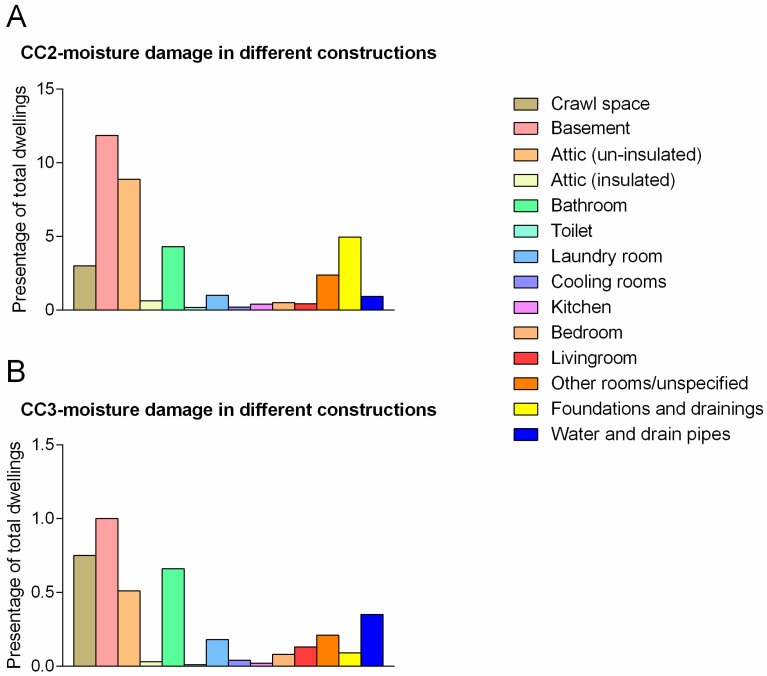
Grade of moisture damage distributed by construction/room type. The figure shows percentage of Norwegian dwellings with CC2 (**A**) and CC3 (**B**) moisture damage in the various rooms.

Approximately 12% of the basements and 9% of the unfurnished attics had CC2 grade. Other rooms and/or constructions with CC2 were drainage (5%), bathrooms (4%) and crawl space (3%), and damages associated with foundation (Figure 5A). CC3 classified damages were most frequently observed in the crawl space, bathroom and unfurnished attic (0.75%, 0.7% and 0.5%, respectively). About 0.35% of the homes had a CC3 associated with water pipes/drainage (Figure 5B).

## 4. Discussion

### 4.1. Representativeness and Biases

The selection of inspected homes in this study did not include homes where the owner initially reported moisture damage. The outcome of the housing check does not affect the price of house insurance, neither is the report used in the event of claims towards the insurance company. Thus, there are no apparent reasons for selection bias within the study population. However, it is important to be aware that the housing check is mainly offered to the insurance company’s best customers, and includes self-owned houses, duplexes, and townhouses only. Our material covers approximately 0.6% of the total amount of this type of dwellings and approximately 0.4% of the total number of dwellings. However, the material is extensive as compared with previous studies in the region and may give a good indication on the prevalence of dampness/moisture problems in the registered type of dwellings.

Since apartment blocks and some other dwelling types such as buildings for shared housing are not surveyed, the study population is not representative for the total Norwegian dwelling stock. Furthermore, the selection of self-owned homes introduces further selection bias, as public housing, cooperative housing, and rented dwellings are not present. Swedish studies have demonstrated that ownership and socioeconomic status are important determinants for self-reported building and indoor air problems; tenants and economically challenged people report more problems than people owning their homes and having higher socioeconomic scores [10,11,12]. In addition, inquiries to NIPH regarding moisture problems tend to be dominated by issues related to rental apartments. These are not covered in the present material.

While some of the observed differences in such studies may be due to a higher tendency to report problems that are the responsibility of others, it is unlikely that such differences in reporting explain all of the differences. Thus, it is highly possible that our study underestimates the proportion of buildings with moisture damage and risk in the total dwelling stock.

### 4.2. Regional Differences

There are striking differences between regions in our material. This could have several possible explanations. One obvious possibility would be climatic differences, as there are great local and regional differences in temperature, precipitation, and wind in Norway. However, it seems somewhat suspect that the Hordaland region, with normal annual precipitation typically exceeding 2100 mm, has a considerably lower than average proportion of moisture damage, while South Trøndelag (Sør Trøndelag) with normal annual precipitation typically well below 1000 mm has the highest rate of moisture problems in this study.

Another explanation might be differences between high-income and low-income regions, but it is unlikely that this is a major explanation, as the two economically quite different regions of Akershus and Oppland county have almost identical proportion of moisture problems, while average area-normalized house prices varies more than twofold between the regions. Oslo, having the highest dwelling prices in the country is very close to the average proportion of moisture damages.

Since a limited group of trained inspectors that were working locally performed the surveys, any systematic differences in scoring between individual surveyors, could have led to apparent regional differences. It seems likely that such differences are present, despite efforts to harmonize the scoring between surveyors.

### 4.3. Building Age

The results indicate a continuous decline in moisture damage and moisture risk from the 1960s to present with a sharper decline from the 1980s. Several distinct factors may have contributed to this.

First, different types of damage caused by malfunction due to wear and tear, or the accumulation of damage from randomly distributed events, would exhibit such a pattern of increasing number of problems with age of the building.

Second, improved building practices, including new and better materials, may have contributed to the decline in problems. Certainly, the use of crawlspaces and uninsulated attics has diminished since the 1980s, and replacing such, high-risk construction with less error-prone alternatives has contributed to the relative scarcity of moisture problems in newer dwellings. Another example of a change in building practice coinciding with the decline in moisture damage is an increasing focus on building airtightness. Inadequate airtightness increases both the risk of condensation outside of the thermal insulation in walls and floors, and moisture intrusion into insulated constructions due to heavy rainfall.

Third, it is conceivable that some of the decline in reported moisture problems could be caused by the lack of their detection in newer buildings. It is probably a general tendency that moisture problems can more easily be detected in older, more simply built houses as compared with more recently built houses. Following the 1970s’ oil crisis, there has been a steadily increasing demand for higher energy efficiency. To achieve this, floors, walls, and roofs facing the outside have become more heavily insulated. This has almost eliminated the risk of condensation on internally exposed surfaces, which would have been readily detected by visual inspection. Simultaneously, the risk of hidden moisture and mold damage may have increased, due to the slower drying out of more heavily insulated constructions. Hidden moisture damage is by definition harder to detect. Uninsulated vs. insulated lofts is an obvious example of constructions where damages are much easier to detect in the former, which is more common in older houses. 

For dwellings from the 1960s and older, the occurrence of moisture damage were surprisingly stable just below 50%.

### 4.4. Location and Type of Moisture Problems

It is clear from Figure 3 that moisture problems were mostly present in rooms typically not occupied for extended periods: basements, uninsulated attics, bathrooms, and crawl spaces. All of these rooms can be exposed to well known risk factors: water intrusion from ground, convection of indoor air with high absolute moisture, intrusion of rain, water leakage from plumbing, and water intrusion in wet zones in bathrooms. Thus, the results are in accordance with what to expect in relation to type of room and potential risk factors. In addition, a significant percentage of refrigerated/cooling rooms had moisture problems, but these rooms were not common in our material.

Interestingly, 15% and 3% of inspected uninsulated and insulated/furnished attics, respectively, had moisture problems. The difference between insulated and un-insulated attics is striking. This could be due to greater difficulties in detecting moisture damage when the attic has been insulated and internally lined. Thus, there could be more hidden moisture damages in insulated attics. However, we are not able to determine this based on the present material.

### 4.5. Comparison with Other Studies

The results for South Trøndelag (area code 70–75) is almost identical with the numbers from the previous study of 205 dwellings in the city of Trondheim (capitol of Sør-Trøndelag) where 50% of the homes had visible signs of moisture problems [7]. Whether this is coincidental remains unclear. Overall, the proportion of homes with moisture damage in the present study was around 30%, which is in agreement with findings from a previous Nordic health survey that reported 27% with moisture problems during an 8-year period [8].

Holme and coworkers reported at least one indicator of moisture problems in 11% of the examined children’s bedrooms [7]. This is in contrast to our present findings, where there was a very low percentage of homes with moisture damage (CC2 or CC3) in bedrooms (1%). This difference may partly be due to Holme and coworkers whom reported moisture in the children’s bedroom, whereas Anticimex inspectors are looking at bedrooms in general. Furthermore, it seems to be differences in what is registered as moisture damage. Holme and coworkers registered moisture damage in children’s bedrooms in the form of stains, swelling, and capillary water extractions in woodwork (2%), condensation on the windows (3%), or condensation on other indoor surfaces (6%). The definition of damage by Anticimex seems narrower, with CC2 involving more serious damage and a need for action, or there is short remaining service life or the building component, whereas CC3 implies total functional failure. Notable is also the huge difference in the sample size between the two studies.

### 4.6. Health Risks

Several large epidemiological studies have shown a consistent correlation between residing in houses with moisture damage or mold inside and respiratory-related disease outcomes, including asthma development and worsening of existing asthma, shortness of breath, coughing, wheezing, respiratory infections, chronic bronchitis, allergic rhinitis, and other symptoms of upper respiratory tract as well as eczema [9,13,14,15,16,17,18,19]. The adverse health effects seem to be of both allergic and non-allergic character. Overall, it has been estimated that there are a 30–50% increase in respiratory problems associated with moisture-related risk factors in homes [20]. This indicates that moisture related risk factors might significantly contribute to the development and/or exacerbation of respiratory disease in the population.

Recent studies indicate that moisture and mold damage in the primary living areas (bedroom, living room, kitchen, bathroom, etc.) are of higher significance for the development of adverse health effects, than moisture and mold damage in rooms that are less used to stay in (e.g., unfurnished basements and attics) [1,2]. Presumably, also larger, more serious moisture and mold damages increase the risk of developing or worsening health problems and disease [3,4,5].

A large proportion of reported moisture damage in our study appears to occur in parts of the dwelling not used for extended or permanent stay. This does not rule out potentially harmful exposure, since pollution components (fungal spores or fragments, bacteria, volatile organic compounds, feces from arthropods, etc.) from the contaminated areas may be spread to other parts of the dwelling. Still, it seems highly unlikely that all of the moisture damage recorded will present identical or any risk of harmful effects. More information on risk differences between different types of moisture damage would be valuable when making decisions about building design, construction procedures, and maintenance. To obtain such information it would probably be necessary to combine methods similar to those described in the present study, with detailed examination of exposure parameters as well as health effects.

### 4.7. Concluding Remarks

The present study shows that around 30% of the inspected dwellings had moisture damage. This is in close agreement with findings from a previous Nordic health survey. When considering the large number of dwellings included in our study, and the thorough inspection protocols of Anticimex, we believe this is a representative estimate of the extent of moisture damage in Norwegian housing. However, as this inspection is primarily offered to the best customers of the If insurance company, and since rental housings are not included, there is a risk that we may underestimate the extent of moisture damage in Norwegian dwellings. Nevertheless, a relatively large proportion of the investigated dwellings had moisture damage. Thus, it is conceivable that this may represent a risk factor for the development or exacerbation of respiratory disease in a relatively large number of individuals, even if only the more extensive moisture damages, and those located in rooms were occupants spend the majority of their time, would have a significant impact on adverse health effects. We must also consider the possibility that climate changes with more rainfall may increase the number of dwellings with moisture problems and associated adverse health effects. Thus, problems with moisture and water damage should be remediated as soon as possible, whereas new buildings must be planned and built in a way that reduces the risk for such problems to occur. In brief, this includes avoiding building constructions that present a risk for water intrusion and excessive moisture accumulation [9]. Adequate ventilation relative to the dampness produced in the house must be ensured, and building structures that can facilitate condensation should be avoided. During the construction period, it is necessary that the building materials are not getting wet and subsequently used in the construction. Present problems with moisture and water damage should be remediated as soon as possible [9]. In this context, it is important to identify and remove the cause of dampness or water accumulation. If this is not taken care of, then the problems are likely to persist. In the case of a leakage, the damaged area must be opened as soon as possible and the area dried, primarily by ventilation and dehumidifiers. Drying out by increasing the temperature should be avoided as this may provide good conditions for mold growth. Mold growth should be removed either by removing damaged materials or by being mechanically cleaned. However, in the event of moisture damage and mold growth, it is difficult to provide completely general and detailed advice covering all situations, and an evaluation of the extent of the measures should be performed in each case. Health-relevant remedial measures can be significantly more extensive than what are often suggested from a building-based assessment of the damage.

## 5. Conclusions

This inspector based survey of more than 10,000 self-owned dwellings in Norway (not including apartments, flats, or rental housing) shows that approximately 30% of the checked dwellings had some type of moisture damage. The number of dwellings with moisture damage varied with the region and age of the building. The observed damage was mostly present in rooms typically not occupied for extended periods such as basements, uninsulated attics, bathrooms, and crawl spaces. Due to the high numbers of dwellings inspected, the estimated numbers are likely to be representative for these type of dwellings, although there is a possibility for some underestimation when taking into account the total number of dwellings in Norway, especially since rented housing, where there are often a number of complaints, is excluded from the survey.

## Figures and Tables

**Table 1:** Definition of condition grades (CCs) used in the building inspection reports.

**CC0**	No remarks (faultless). Documentation of professional execution/correct construction, including material use and technical solutions is presented when necessary.
**CC1**	As CC0, but the building part/construction has minor wear without need of measures.
**CC2**	The building part is not constructed correctly, is damaged (or has symptoms of damage), is considerably worn, or with reduced function. Measures are needed or there is short remaining life expectancy. May also include lack of documentation of correct construction of structures or building parts at particular risk of moisture damage.
**CC3**	Total malfunction. The building part/construction does no longer fulfill its purpose, or deviates from building regulations/rules. Need for urgent measures.
